# Decreased retinal nerve fiber layer thickness in patients with cerebral venous thrombosis

**DOI:** 10.1186/s12886-019-1046-9

**Published:** 2019-02-20

**Authors:** Yaran Koban, Hatice Ozlece, Sibel Karayol, Nergiz Huseyinoglu

**Affiliations:** 1 Faculty of Medicine, Department of Ophthalmology, University of Kafkas, Merkez, 36100 Kars, Turkey; 2 Department of Neurology, Acıbadem Kayseri Hospital, Kayseri, Turkey; 3 Faculty of Medicine, Department of Radiology, University of Harran, Sanliurfa, Turkey

**Keywords:** Axonal loss, Cerebral venous thrombosis, Macular thickness, Optical coherence tomography, Retinal nerve fiber layer

## Abstract

**Background:**

To identify thickness variations in the retinal nerve fiber layer around the optic disc and macula in patients with cerebral vein thrombosis (CVT) without papilledema.

**Methods:**

This study included 28 patients with CVT diagnosis and appropriate treatment. Detailed ophthalmologic examination found bilateral vision 10/10, vision field test normal and fundus examination found no papilledema images. The patients had macular and optic retinal nerve fiber layer thickness (RNFL) measured with spectral domain-optical coherence tomography (SD-OCT) (Optovue, Fremont, CA). Patients had retinal nerve fiber thickness compared with a control group.

**Results:**

When the effect on the macula and RNFL near the optic nerve disk is investigated, there was significant thinning identified in the macula inferior inner, temporal inner, superior inner and temporal outer quadrants (*p* = 0.009, 0.001, 0.026, 0.014, respectively) and in the inferior temporal quadrant of the optic nerve disk (*p* = 0.020) in CVT patients compared to normal individuals.

**Conclusions:**

Even after appropriate treatment of CVT patients, axonal loss was identified with OCT. As a result, it may be important to use OCT measurements to monitor CVT treatment.

## Background

Thrombosis of the cerebral veins and sinuses (CVT) is a rare cerebrovascular disease, that may become significant with missed or late diagnosis. It may be observed in all age groups, more commonly in very young and middle-aged individuals and mainly affects women.^1^ Though pregnancy, puerperium, oral contraceptive use, coagulopathies, intracranial infections, cranial tumors, penetrant head trauma, lumbar puncture, malignancy, dehydration, inflammatory bowel disease, connective tissue diseases, Behçet disease, sarcoidosis, nephrotic syndrome, parenteral infusions and a variety of medications have been shown among causes, very detailed investigations have not determined a cause in 20–25% of cases [1–4].

Visual disorders linked to the increase in intracranial pressure like papilledema, diplopia and blurred vision may accompany the tableau [5, 6]. However, the effect of CVT on the eye in the long term continues to be uncertain. The aim of this study is to assess optic disc retinal nerve fiber and macula thickness variations in patients with CVT without papilledema after appropriate treatment. To the best of our knowledge, the present study is the first to evaluate the posterior ocular segment changes in patients with CVT using optical coherence tomography (OCT) or traditional imaging modalities.

## Materials and methods

This prospective case-control study, which was conducted by the Department of Ophthalmology and Department of Neurology, Kafkas University Medical Faculty Hospital (KUMFH), adhered to the tenets of the declaration of Helsinki and was approved by the local ethics committee (80576354–050-99/122; *Kafkas University, Human Ethics Comitteee, Kars, Turkey*). Our study included all consecutive patients over the age of 18 treated in the KUMFH area for CVT between December 2012 and July 2016. All subjects provided informed consent to participate in the study.

### Participants

**Fig. 1:**
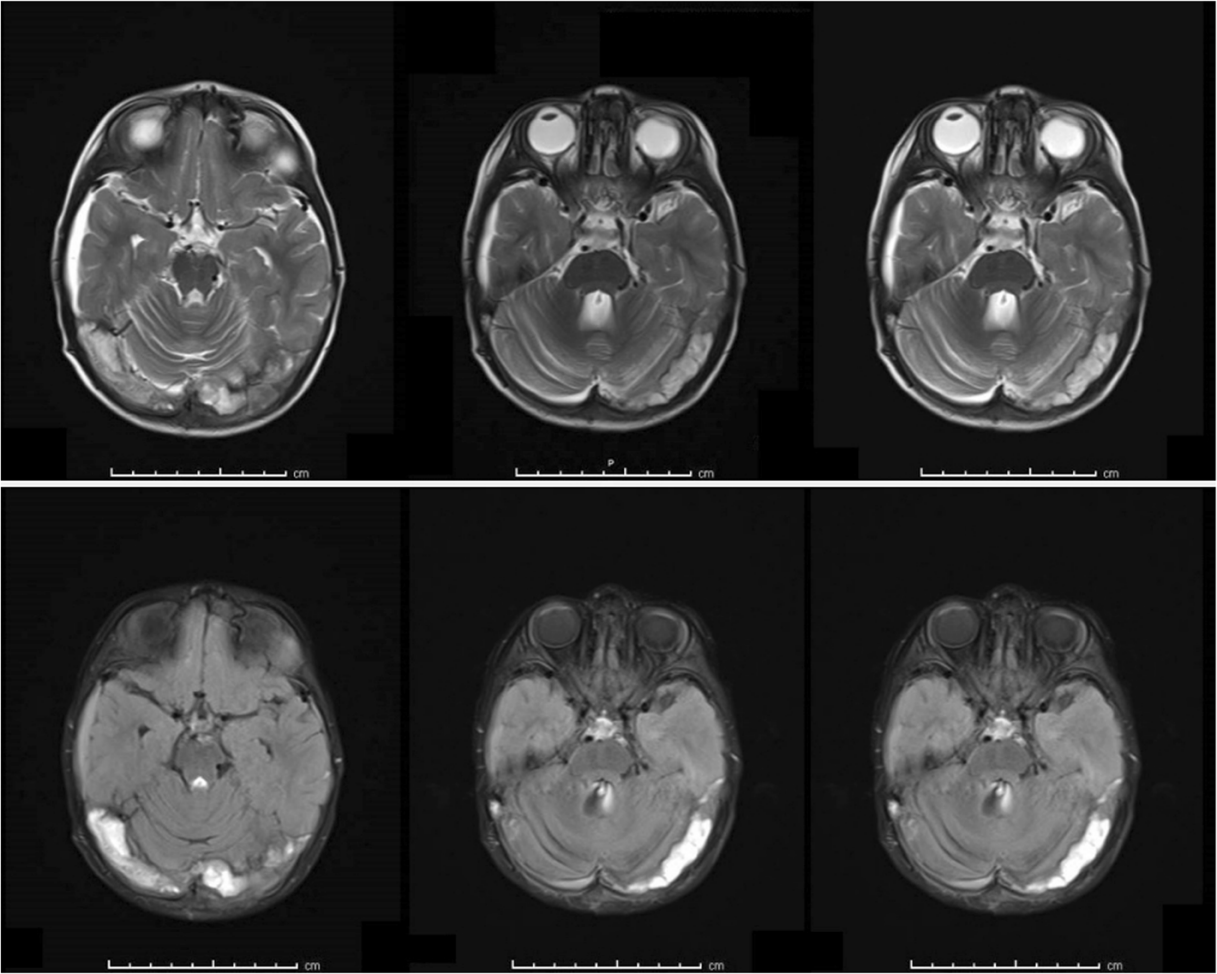
Increased signal intensity on T1, T2 and FLAIR-weighted series compatible with thrombus in right and left transverse sinuses

The study included 28 patients with CVT diagnosis at Kafkas University Medical Faculty monitored in the Neurology clinic with best improved vision sharpness on Snellen eye chart and 30 healthy volunteers with examination at the ophthalmology clinic. The 28 CVT patients had been admitted to the Kafkas University Medical Faculty Neurology clinic, with definite diagnosis with cranial magnetic resonance imaging (MRI) and MR venography and had common complaints of severe headaches previously unknown or with changed character. The majority of patients had vomiting complaint. Five patients applied to the hospital due to experiencing epileptic seizures. None of the patients had vision complaints. During admission to the neurology ward, patients had consciousness varying from open to stupor, with neurologic deficit present in some patients. Seven patients had radiological diagnosis of thrombosis mainly of the right sinuses (sigmoid, transverse, v. jugularis), with 21 diagnosed with thrombosis of the left sinuses. Additionally, on radiology some patients had thrombosis of the central sinuses (superior and inferior sagittal, sinus rectus, confluence sinus) (Fig. 1). All patients had anticoagulant treatment while some had antiedema treatment administered. On first neurological examination, no patient had papilledema.

For the study, patients had ophthalmologic examination when general state was stable, when neurological symptoms regressed and after antiedema treatment was stopped. Ophthalmologic examination was completed from 9 to 12 months after the patients applied to the neurology clinic. During ophthalmologic examination, all patients were conscious and had no neurological symptoms.

The selection criteria were normal optic nerve appearance on dilated stereoscopic examination and fundus photography; normal visual field defect at Humphrey perimeter during follow up. None of the patient and control group had thyroid disorders, hypertension, diabetes mellitus, cardiovascular events, Cushing disease, or congenital adrenal hyperplasia. Patients and healthy volunteers who used alcohol or tobacco were excluded from the study. The patients and control subjects who underwent ocular surgery or had ocular trauma or any ocular diseases at the time of OCT measurement were excluded as well. The control group consisted of normal healthy volunteers recruited from among the hospital staff.

### Study protocol

The diagnosis of CVT had to be confirmed by cerebral MRI combined with cerebral MR venography, following established diagnostic criteria [7]. Each patient underwent a comprehensive ophthalmologic examination. Following this detailed ophthalmologic examination, macular and RNFL thickness were measured using spectral domain OCT with digital software (RTVue-100, Optovue Inc., Fremont, CA, USA). OCT imaging was performed through undilated pupils and with the same intensity of dim room lighting. All images were taken by the same operator. MM6 (12 radial line scans with 1024 A-scans each, within 6 mm diameter) protocol was used to detect the macular thickness measurements. The scan was divided into the nine Early Treatment Diabetic Retinopathy Study (ETDRS) subfields [8]. The parameters registered in this study were superior outer macular thickness (SOM), inferior outer macular thickness (IOM), temporal outer macular thickness (TOM), nasal outer macular thickness (NOM), superior inner macular thickness (SIM), inferior inner macular thickness (IIM), temporal inner macular thickness (TIM), nasal inner macular thickness (NIM), and central foveal thickness (CFT). The peripapillary RNFL thickness and parameters were calculated by fast RNFL scan, which measures RNFL thickness at 3.45 mm from the center of the optic disc.

### Statistics

SPSS 11.5 (SPSS Inc., USA) software was used to perform the statistical analyses. The Mann-Whitney U test was used to compare numeric variables between headache and control groups and *P* values < 0.05 were considered as statistically significant.

## Results

The mean age of 28 CVT cases with ages from 21 to 36 was 27.72 ± 5.12 years, while the mean age of 30 cases in the control group with ages from 18 to 43 was 29.83 ± 9.02 years. Of CVT cases 3 were male (10.7%) and 25 (89.3%) were female. The control group included 5 males (16.7%) and 25 females (83.3%). The mean axial length of patients in the CVT group was 23.9 ± 0.91, while for healthy volunteers this was 23.9 ± 0.84. There was no significant difference in age, gender distribution and axial length between the 2 groups (*p* = 0.116, p = 0,492, *p* = 0.457, respectively). Detailed ophthalmologic examination of patients and volunteers in the control group found bilateral vision 20/20, with mean intra ocular pressure in the CVT group 18.0 ± 0.2 mmHg and in the healthy volunteers 16.9 ± 2.0 (*p* = 0.214). Bilateral anterior segment findings were natural. Fundus examination found no papilledema images and visual field tests were normal in both eyes.

### Comparison of macular thickness in patients with CVT and healthy volunteers

We found that all macular thickness parameters were lower in eyes with CVT versus to normal eyes. This thinning in the values was significant for IIM, TIM, SIM, and TOM segments (*p* = 0.009, *p* = 0.001, *p* = 0.026, *p* = 0.014, respectively) *(*Table 1*).* In terms of central, parafoveal and perifoveal macular thickness parameters, there was no statistically significant difference identified between the right and left eyes of CVT patients (*p* = 0.025).

**Table 1:** Results of SD-OCT Macular Thickness (mm) parameters

Parameters	SVT group Mean SD	Control group Mean SD	p
Fovea thickness (mm)	238.44 ± 19,89	245.90 ± 28.57	0. 263
Inferior inner macula (mm)	300.89 ± 28.93	328.67 ± 45.37	**0. 009**
Temporal inner macula (mm)	296.33 ± 13.30	309.17 ± 12.02	**0. 001**
Superior inner macula (mm)	311.96 ± 14.90	333.33 ± 46,29	**0. 026**
Nasal inner macula (mm)	304.37 ± 57.51	323.17 ± 12.41	0. 086
Inferior outer macula (mm)	267.41 ± 38.80	284.27 ± 43.59	0.130
Temporal outer macula (mm)	256.15 ± 32.22	272.50 ± 13.50	**0.014**
Superior outer macula (mm)	276.56 ± 11.92	290.13 ± 45.31	0.137
Nasal outer macula (mm)	290.89 ± 13.47	298.10 ± 15.06	0.063

*SVT Sinus venous thrombous, SD Standart deviation; p:*

Significant differences at *p* < 0.05 are shown in bold face

### Average Peripapillary RNFL thickness in patients with CVT and healthy volunteers

We found that inferior temporal (IT) part of the peripapillary RNFL were significantly thinner in the CVT group versus to those in the healthy control group (*p* = 0.020). There was no significant difference in superior nasal (SN), nasal upper (NU), nasal lateral (NL), inferior nasal (IN), temporal lateral (TL), temporal upper (TU), and superior temporal (ST) scores between the 2 groups. Table 2 shows sectoral RNFL measurements for patients with CVT and controls. For the right and left eyes of CVT patients, there was no statistically significant difference identified for values for the same peripapillary segments (*p* = 0.25).

**Table 2:** Results of SD-OCT RNFL Thickness (mm) parameters

RNFL	SVT group Mean SD	Control group Mean SD	p
ST	153.65 ± 25.27	149.07 ± 20.23	0. 439
SN	124.45 ± 26.91	121.43 ± 20.92	0. 627
TU	90.19 ± 17.16	86.83 ± 13.54	0. 399
NU	83.13 ± 13.79	83.90 ± 13.11	0. 345
TL	81.71 ± 15.06	82.17 ± 11.44	0. 895
NL	76.00 ± 12.32	73.53 ± 11.77	0. 427
IT	146.06 ± 15.03	156.63 ± 10.21	**0. 020**
IN	120.68 ± 23.66	124.43 ± 23.22	0. 534

*RNFL Retinal nerve fiber layer, ST Superior temporal, SN Superior nasal, TU Temporal upper, NU Nasal upper, TL Temporal lateral, NL Nasal lateral, IT Inferior temporal, IN Inferior nasal, SVT Sinus venous thrombous, SD Standart deviation; p*

Significant differences at *p* < 0.05 are shown in bold face

## Discussion

This study investigated the macula and optic nerve disc RNFL thicknesses in patients with previous CVT diagnosis and treatment, with no vision complaints or papilledema at time of examination and identified that there was significant thinning in the IIM, TIM, SIM and TOM segments of the macula and the IT sector of the optic nerve disc compared to normal individuals. In light of these results, we conclude that after even effective treatment, axonal loss can be detected by OCT in CVT patients. We consider this retinal axonal loss may have developed as a result of subclinical increased intracranial pressure and chronic papilledema not identified by ophthalmoscopy.

Onset forms and clinical symptoms of CVT is extraordinarily variable, and such variability depends on different factors, such as the location, size, duration, and rapidity of development of thrombus. Obstruction of the intracranial venous drainage system secondary to a thrombus is most commonly observed in the superior sagittal sinuses (70–80%). Later transverse, sigmoid and at lower rates cavernous sinuses are affected [9]. In one third of cases, more than one sinus is affected [10]. Patients may apply with symptoms linked to increased intracranial pressure (headache, papilledema), focal neurological deficits, seizures, encephalopathy or tableau associated with these situations. Elevated opening pressure is a frequent finding in CVT and is present in > 80% of patients. Headache is the most common symptom of CVT considered to occur linked to intracranial pressure, and is present in approximately 90% of cases. The most common onset symptom of headache in 70–75% and benign intracranial hypertension seen in 20–40% of patients may be observed as a single symptom [6].

Eye symptoms may accompany the tableau 30.5% of the time and is observed as a single onset presentation in 21.2% of all CVT patients. The chief abnormal ocular complaint for 85.9% is blurring and degeneration of acute vision. It is argued that underlying ocular symptom of CVT are venous obstruction and intracranial hypertension [11]. CVT patients exhibit not only vision loss, metamorphopsia, and diplopia, but also the symptoms of eyeball movement restriction, orbital pain, exophthalmos, chemosis, nystagmus, visual area defect and fundus changes. The symptoms of fundus changes include papilledema, optic atrophy, optic disc hemorrhage, retinal hemorrhage, retinal varices, and macular edema [11, 12]. The most common symptom in eyes is papilledema [11]. Studies have reported papilledema in 27–68.7% of CVT cases [10, 11, 13].

The disc swelling in papilledema is primarily due to a rise of intracranial pressure (ICP) which produces axoplasmic flow stasis in the optic nerve head. Visual loss from papilledema can occur even in cases of mild ICP elevation. Elevated ICP causes a number of effects on the visual system, the most severe of which is nerve fiber dysfunction from swelling, progressive loss of retinal ganglion cell somas in the retina and retinal ganglion cell axons within the optic nerve, and optic atrophy [14]. In our study, as in previous studies of CVT patients, papilledema diagnosis was made with ophthalmoscopic examination. None of our patients had papilledema identified, but even after appropriate treatment, compared with normal controls, all patients had significantly reduced macular and RNFL thickness. The cause of this thinning was not assessed with ophthalmoscope at time of application, but may be subclinical papilledema and/or subclinical ICPIS.

Monteiro and Afonso evaluated 52 eyes of patients with resolved chronic papilledema from idiopathic intracranial hypertension. Their results showed significantly reduced macular and RNFL thickness and confirmed the ability of OCT to quantify axonal loss in this condition [15]. They reported that this reduction might be explained by transient ischemia due to intra- and extraaxonal edema resulting in atrophy of the retinal nerve fibers. Their study showed that axonal loss following papilledema can be estimated by OCT-measured macular thickness measurements. In eyes with resolved papilledema demonstrated significantly decreased macular thickness in eight out of nine macular parameters compared to the control group, while OCT-measured RNFL thickness were thinner in six of seven RNFL thickness parameters. In our study, we identified significant thinning in 4 of the 9 macular parameters and 1 of the 8 RNFL parameters assessed in CVT patients. We think the cause of this difference may be still present subclinical ICPIS and/or incomplete resolution of papilledema during OCT measurements.

Coagulation mechanisms are blamed for occurrence of CVT. As a result, anticoagulants are primarily used for CVT treatment [16, 17]. In addition to anticoagulants forming the basis of treatment, symptomatic treatment is important. Especially in the acute period antiedema treatment to prevent intracranial hypertension that may occur during clinical progression of the disease may be important. Patients should be monitored carefully for intracranial hypertension and complications; antiedema treatment duration should be set according to clinical assessment, fundus examination and vision field tests [18]. All cases had treatment arranged according to the guidelines and patients were monitored closely for intracranial hypertension. None had papilledema or vision field defects identified. In spite of this, the RNFL and macular layer thinning identified in our study shows that OCT is important for monitoring CVT patients and deciding when to stop antiedema treatment. Because OCT makes it possible to detect mild papilledema that could not be visualized using traditional methods [19, 20].

A limitation of the present study is that some patients were on anti-edema treatment. Although, we determined that macular thickness parameters were lower in nearly all eyes with CVT, there might be patients with high intracerebral pressure which might affect the RNFL thickness.

## Conclusions

This study was, to the best of our knowledge, the first evaluation of the macular and peripapillary RNFL thickness changes in CVT patients. In conclusion, when patients with no papilledema on fundus examination, with no visual symptoms or vision field defects are assessed with the more sensitive method of OCT, thinning of macula and RNFL thicknesses was observed. All patients with CVT diagnosis should have detailed eye examination including OCT investigation at time of application and during monitoring. Thus, mild papilledema at time of application and macula thickness changes during check-ups will be identified so treatment and monitoring of patients may be completed with more effective methods and eye and cerebral complications will be prevented. We believe our study will be important for monitoring and treatment planning for patients with CVT accompanied by diseases affecting the optic nerve disc like glaucoma.

## Abbreviations

CFT: Central foveal thickness
CVT: Cerebral vein thrombosis
IIM: Inferior inner macular thickness
IOM: Inferior outer macular thickness
NIM: Nasal inner macular thickness
NOM: Nasal outer macular thickness
OCT: Optical coherence tomography
RNFL: Retinal nerve fiber layer thickness
SIM: Superior inner macular thickness
SOM: Superior outer macular thickness
TIM: Temporal inner macular thickness
TOM: Temporal outer macular thickness
